# Activation of NF-κB by the RANKL/RANK system up-regulates snail and twist expressions and induces epithelial-to-mesenchymal transition in mammary tumor cell lines

**DOI:** 10.1186/1756-9966-32-62

**Published:** 2013-09-05

**Authors:** Masanobu Tsubaki, Makiko Komai, Shin-ichiro Fujimoto, Tatsuki Itoh, Motohiro Imano, Kotaro Sakamoto, Hirotaka Shimaoka, Tomoya Takeda, Naoki Ogawa, Kenji Mashimo, Daiichiro Fujiwara, Junji Mukai, Katsuhiko Sakaguchi, Takao Satou, Shozo Nishida

**Affiliations:** 1 Division of Pharmacotherapy, Kinki University School of Pharmacy, Kowakae, Higashi-Osaka 577-8502, Japan; 2 Department of Pathology, Kinki University School of Medicine, Osakasayama, Osaka, Japan; 3 Department of Surgery, Kinki University School of Medicine, Osakasayama, Osaka, Japan; 4 Department of Pharmacy, Izumi Municipal Hospital, Izumi, Osaka, Japan; 5 Department of Pharmacy, Japanese Red Cross Society Wakayama Medical Center, Wakayama, Japan

**Keywords:** RANK, RANKL, EMT, Breast cancer, NF-κB

## Abstract

**Background:**

Increased motility and invasiveness of cancer cells are reminiscent of the epithelial-mesenchymal transition (EMT), which occurs during cancer progression and metastasis. Recent studies have indicated the expression of receptor activator of nuclear factor-κB (RANK) in various solid tumors, including breast cancer. Although activation of the RANK ligand (RANKL)/RANK system promotes cell migration, metastasis, and anchorage-independent growth of tumor-initiating cells, it remains to be investigated if RANKL induces EMT in breast cancer cells. In this study, we investigated whether RANKL induces EMT in normal breast mammary epithelial cells and breast cancer cells, and the mechanism underlying such induction.

**Methods:**

Expression levels of vimentin, N-cadherin, E-cadherin, Snail, Slug, and Twist were examined by real-time polymerase chain reaction. Cell migration and invasion were assessed using Boyden chamber and invasion assays, respectively. The effects of RANKL on signal transduction molecules were determined by western blot analyses.

**Results:**

We found that stimulation by RANKL altered the cell morphology to the mesenchymal phenotype in normal breast epithelial and breast cancer cells. In addition, RANKL increased the expression levels of vimentin, N-cadherin, Snail, and Twist and decreased the expression of E-cadherin. We also found that RANKL activated nuclear factor-κB (NF-κB), but not extracellular signal-regulated kinase 1/2, Akt, mammalian target of rapamycin, c-Jun N-terminal kinase, and signal transducer and activator of transcription 3. Moreover, dimethyl fumarate, a NF-κB inhibitor, inhibited RANKL-induced EMT, cell migration, and invasion, and upregulated the expressions of Snail, Twist, vimentin, and N-cadherin.

**Conclusions:**

The results indicate that RANKL induces EMT by activating the NF-κB pathway and enhancing Snail and Twist expression. These findings suggest that the RANKL/RANK system promotes tumor cell migration, invasion, and metastasis via the induction of EMT.

## Background

Breast cancer remains the most common cancer among women worldwide [1]. Although treatment of early stage breast cancer by surgical resection and adjuvant therapy has a good prognosis, the development of metastatic breast cancer is responsible for the majority of cancer-related mortality. Advanced breast cancer commonly spreads to the bone, lung, liver, or brain, with bone and lung being the most common sites of breast cancer metastasis. Almost all patients with advanced breast cancer eventually develop metastases. Therefore, understanding the mechanisms that facilitate metastasis is of importance.

The epithelial-mesenchymal transition (EMT) is a common phenotypic transformation in cancer cells that causes loss of cell-cell adhesion and increases cell motility [2-4], thereby increasing their metastatic potential. Downregulation of E-cadherin expression is possibly the most important consequence of EMT that leads to the changed behavior of cancer cells [5,6]. An important event in EMT is the switching of expression from E-cadherin, which is downregulated, to N-cadherin, which in turn is upregulated [7]. Other mesenchymal proteins, e.g., vimentin, are also upregulated during EMT [8,9]. EMT is regulated by transcription factors such as Snail1, Slug, and Twist that simultaneously induce the expression of genes required for mesenchymal properties and repress the expression of genes that are required for the epithelial phenotype [10]. The expression of EMT-induced transcription factors is controlled at the transcription level by proteins such as NF-κB, β-catenin, and Smad and via the mitogen-activated protein kinase pathway or the phosphoinositol 3-kinase/Akt pathway [11-15].

Receptor activator of NF-κB (RANK) and RANK ligand (RANKL) were originally shown to be essential for osteoclastogenesis, lymph node development, and formation of lactating mammary glands during pregnancy. Recent studies reported the expression of RANK and RANKL in various solid tumors, including breast cancer [16,17]. RANKL accelerates the migration and metastasis of cancer cells expressing RANK [16-18]. In addition, RANKL can protect breast cancer cells from apoptosis in response to DNA damage, as well as control the self-renewal and anchorage-independent growth of tumor-initiating cells [19]. However, it remains to be investigated if RANKL induces EMT in breast cancer cells. Therefore, we investigated whether RANKL induces EMT in normal breast mammary epithelial cells and breast cancer cells, and the mechanism underlying such induction.

## Materials and methods

### Materials

Soluble RANKL was purchased from PeproTech (London, UK). This reagent was dissolved in PBS (0.05 M, pH7.4), and used for various assays described below.

Dimethyl fumarate (DMF) was purchased from Wako (Tokyo, Japan), and dissolved in dimethyl sulfoxide (DMSO). This reagent was dissolved in phosphate buffer saline (PBS; 0.05 M, pH7.4), filtrated through Syringe Filters (0.45 μm, IWAKI GLASS, Tokyo, Japan) and used for various assays described below.

### Cell culture

4T1 and NMuMG cells were provided by American Type Culture Collection (Rockville, MD, USA). MCF-7 cells were obtained from Health Science Research Resources Bank (Osaka, Japan). These cells were cultured in RPMI1640 medium (Sigma) supplemented with 10% fetal calf serum (Gibco, Carlsbad, CA, USA), 100 μg/ml penicillin (Gibco), 100 U/ml streptomycin (Gibco), and 25 mM HEPES (pH 7.4; Wako) in an atmosphere containing 5% CO_2_.

### Evaluation of epithelial-mesenchymal transition (EMT)

4T1, MCF-7, and NMuMG cells were photographed using a light microscope daily to monitor for change in morphology. To determine whether EMT was influenced by RANKL, 4T1, MCF-7, and NMuMG cells were plated on plates coated with gelatin (Sigma, St. Louis, MO, USA) in the presence of maintenance media plus 0 or 100 ng/ml RANKL.

### Quantitative real-time polymerase chain reaction (PCR)

Total RNA was isolated using RNAiso (Takara Biomedical, Siga, Japan). One microgram of purified total RNA was used for the real-time PCR analysis with the SuperScript First-Strand Synthesis System (Invitrogen, Carlsbad, CA). cDNA was subjected to quantitative real-time PCR by using SYBR Premix Ex *Taq* (Takara Biomedical) and the ABI Prism 7000 detection system (Applied Biosystems, Foster, CA) in a 96-well plate according to the manufacturer’s instructions. The PCR conditions for glyceraldehyde-3-phosphate dehydrogenase (GAPDH), Snail, Slug, Twist, Vimentin, N-cadherin, and E-cadherin were 94°C for 2 min; followed by 40 cycles of 94°C for 0.5 min, 50°C for 0.5 min, and 72°C for 0.5 min. The following primers were used: Snail, 5′- GCG AGC TGC AGG ACT CTA AT −3′ (5′-primer) and 5′- GGA CAG AGT CCC AGA TGA GC −3′ (3′-primer); Slug, 5′- CGT TTT TCC AGA CCC TGG TT −3′ (5′-primer) and 5′- CTG CAG ATG AGC CCT CAG A −3′ (3′-primer); Twist, 5′- CGC CCC GCT CTT CTC CTC T −3′ (5′-primer) and 5′- GAC TGT CCA TTT TCT CCT TCT CTG −3′ (3′-primer); Vimentin, 5′- AGA TGG CCC TTG ACA TTG AG −3′ (5′-primer) and 5′- CCA GAG GGA GTG AAT CCA GA −3′ (3′-primer); N-cadherin, 5′- CTC CTA TGA GTG GAA CAG GAA CG −3′ (5′-primer) and 5′- TTG GAT CAA TGT CAT ATT CAA GTG CTG TA −3′ (3′-primer); E-cadherin, 5′- GAA CGC ATT GCC ACA TAC AC -3′ (5′-primer) and 5′- GAA TTC GGG CTT GTT GTC AT -3′ (3′-primer); and GAPDH, 5′-ACT TTG TCA AGC TCA TTT-3′ (5′-primer) and 5′-TGC AGC GAA CTT TAT TG-3′ (3′-primer). As an internal control for each sample, the GAPDH gene was used for standardization. Cycle threshold (Ct) values were established, and the relative difference in expression from GAPDH expression was determined according to the 2^–∆∆Ct^ method of analysis and compared to the expression in control cells.

### Western blotting

#### Preparation of nuclear extracts for NF-κB

4T1 and NMuMG cells treated under various conditions were washed with cold PBS and suspended for 30 min in 0.4 ml of a hypotonic lysis buffer (20 mM Tris–HCl (pH 7.5), 10 mM NaCl, 1 mM EDTA, 2 mM Na_3_VO_4,_) containing protease inhibitors (10 μg/ml leupepton, 1 μM pepstatin). The cells were then lysed with 12.5 μl of 10% nonyl phenoxylpolyethoxylethanol (NP-40). The homogenate was centrifuged, and the supernatant, which contained the cytoplasmic extracts, was stored at −80°C. The nuclear pellet was resuspended in 25 μl of ice-cold nuclear-extraction buffer for 30 min, with intermittent mixing. Then, the extract was centrifuged, and the supernatant containing the nuclear extract was obtained. The protein content was measured by using the BCA protein assay kit (Pierce, Rockford, IL, USA). The nuclear and cytoplasmic extracts (40 μg of protein) were fractionated on polyacrylamide-sodium dodecyl sulfate (SDS) gels and transferred to polyvinylidene fluoride (PVDF) membranes (Amersham, Arlington Heights, IL, USA). The membranes were blocked with a solution containing 3% skim milk and incubated with the anti-NF-κB p65 antibody (Cell Signaling Technology, Beverly, MA, USA) overnight at 4°C. Subsequently, the membranes were incubated with anti-rabbit IgG sheep antibody coupled to horseradish peroxidase (Amersham) for 1 h at room temperature. The reactive proteins were visualized by using ECL-plus (Amersham) according to the manufacturer’s instructions. Anti-lamin A antibody (Santa Cruz Biotechnologies, CA, USA) was used as the internal standard; it was used as the primary antibody to detect lamin A.

#### Preparation of whole-cell lysates

4T1 and NMuMG cells treated under various conditions were lysed with a lysis buffer containing 20 mM Tris–HCl (pH 8.0), 150 mM NaCl, 2 mM EDTA, 100 mM NaF, 1% NP-40, 1 μg/ml leupeptin, 1 μg/ml antipain, and 1 mM phenylmethylsulphonyl fluoride. The protein content in the cell lysates was determined using a BCA protein-assay kit. The extracts (40 μg of protein) were fractionated on polyacrylamide-SDS gels and transferred to PVDF membranes (Amersham). The membranes were blocked with a solution containing 3% skim milk and incubated overnight at 4°C with each of the following antibodies: anti-NF-κB p65, anti-phospho-extracellular signal-regulated kinase (ERK) 1/2 antibody, anti-phospho-Akt antibody, anti-phospho-mammalian target of rapamycin (mTOR) antibody, anti-phospho-c-Jun N-terminal kinase (JNK) antibody, anti-phospho-signal transducers and activator of transcription 3 (STAT3) antibody, anti-ERK1/2 antibody, anti-Akt antibody, anti-mTOR antibody, anti-JNK antibody, and anti-STAT3 antibody (Cell Signaling Technology). Subsequently, the membranes were incubated with horseradish peroxidase-coupled anti-rabbit IgG sheep antibodies (Amersham) for 1 h at room temperature. The reactive proteins were visualized using ECL-plus (Amersham) according to the manufacturer’s instructions. As an internal standard, anti-β-actin mouse monoclonal antibody (Sigma) was used as the primary antibody to detect β-actin protein.

### In vitro migration and invasion assays

Migration was analyzed in a Boyden chamber assay using Falcon cell culture inserts (pore size, 8.0 μm; Becton Dickinson, Franklin Lakes, NJ, USA). Analysis of invasive properties was achieved by using Falcon cell culture inserts covered with 50 μg of Matrigel (Becton Dickinson). For both assays, the upper chamber of the insert was filled with 500 μL of the cell and drug suspension (5×10^3^ cells) and conditioned medium (addition of RANKL in serum-free medium) was added to the lower chamber. After the cells had been incubated for 24 hr, the remaining cells in the upper layer were swabbed with cotton and penetrating cells in the lower layer were fixed with 95% ethanol and removed for hematoxylin staining. Cells passing through the 8 μm-pore culture inserts were counted using light microscopy.

### Statistical analysis

All results are expressed as means and S.D. of several independent experiments. Multiple comparisons of the data were done by ANOVA with Dunnet’s test. *P* values less than 5% were regarded as significant.

## Results

### RANKL promotes the EMT, migration, and invasion of breast cancer cells and normal mammary epithelial cells

**Figure 1:**
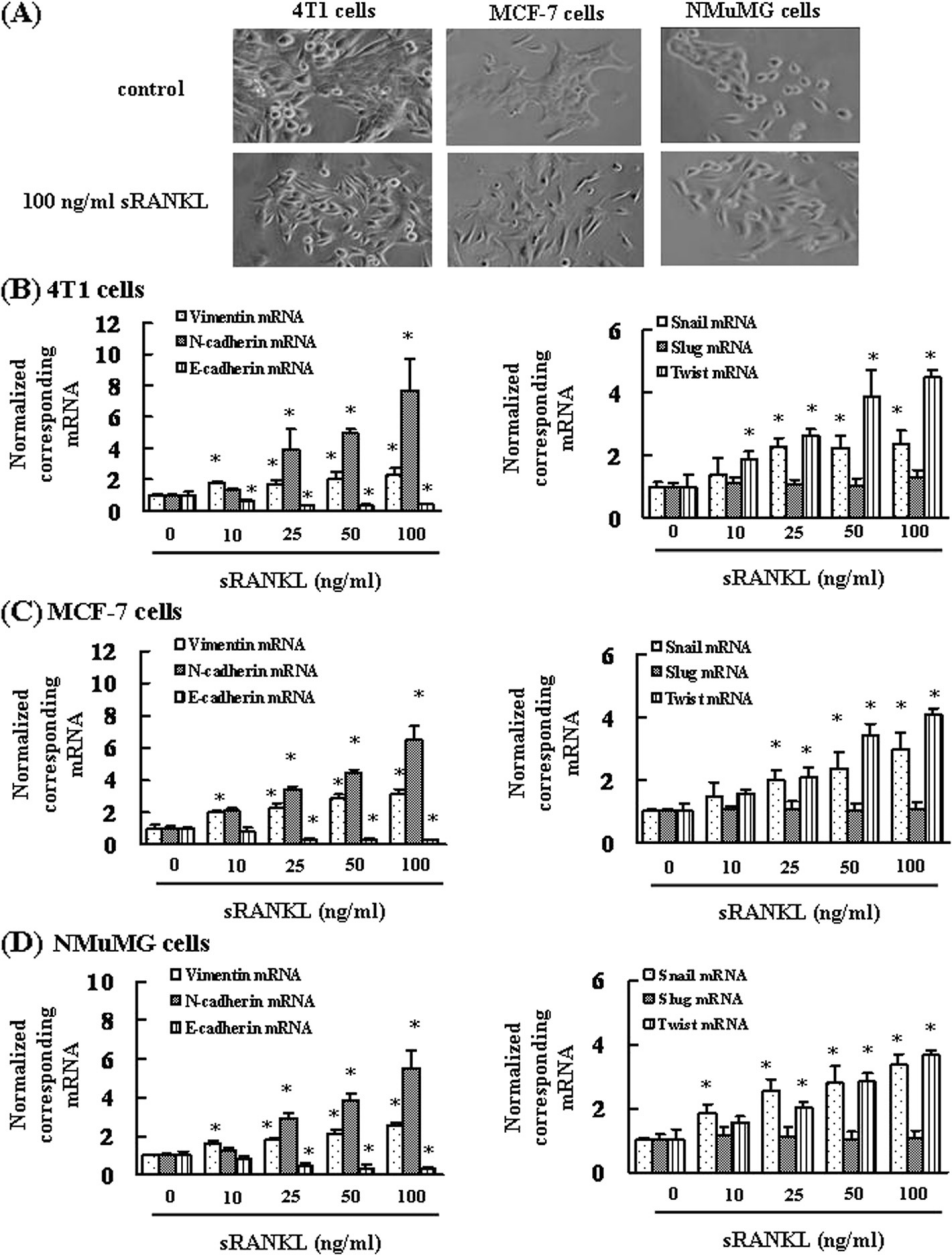
**RANKL enhances Snail and Twist expression and induces changes in the morphology of cells. (A)** Analysis of cell morphology after cell treatment of with 100 ng/mL RANKL. RANKL induces changes in the epithelial morphology of 4T1, MCF-7, and NMuMG cells (×40 magnification). **(B-D)** Total RNA was extracted, and the mRNA expression levels of vimentin, E-cadherin, N-cadherin, Snail, Slug, and Twist were determined by real-time PCR. The results are expressed as treated over control ratio after correction to GAPDH mRNA levels. The results are representative of 5 independent experiments. *p < 0.01, as compared to controls (ANOVA with Dunnett’s test).

In order to determine the induction of EMT by RANKL in breast cancer cells, we investigated the change in morphology following stimulation with RANKL. After 48 h of treatment, the morphology of 4T1, MCF-7, and NMuMG cells changed from an epithelial sheet-like structure to a mesenchymal fibroblastic spindle shape, which is characteristic of EMT (Figure 1A). We also found that these cells expressed RANK (data not shown). Next, in order to investigate the molecular mechanism of RANKL-mediated EMT of breast cancer cells and normal mammary epithelial cells, we examined the effects of RANKL on EMT markers. RANKL stimulation resulted in downregulation of the mRNA of the epithelial marker E-cadherin and upregulation of the mRNAs of the mesenchymal markers vimentin and N-cadherin in a concentration-dependent manner in 4T1, MCF-7, and NMuMG cells (Figure 1B–1D). The expression levels of the transcriptional repressors of E-cadherin, Snail and Twist, were upregulated by RANKL treatment in 4T1, MCF-7, and NMuMG cells (Figure 1B–1D). However, no significant change in the level of Slug mRNA was detected in RANKL-treated cells as compared to control cells in 4T1, MCF-7, and NMuMG cells (phosphate-buffered saline-treated cells) (Figure 1E–1G). In addition, small interfering RNA-mediated silencing of RANK expression suppressed RANKL-induced upregulation of vimentin, N-cadherin, Snail, and Twist mRNAs and RANKL-mediated downregulation of E-cadherin mRNA (data not shown).

**Figure 2:**
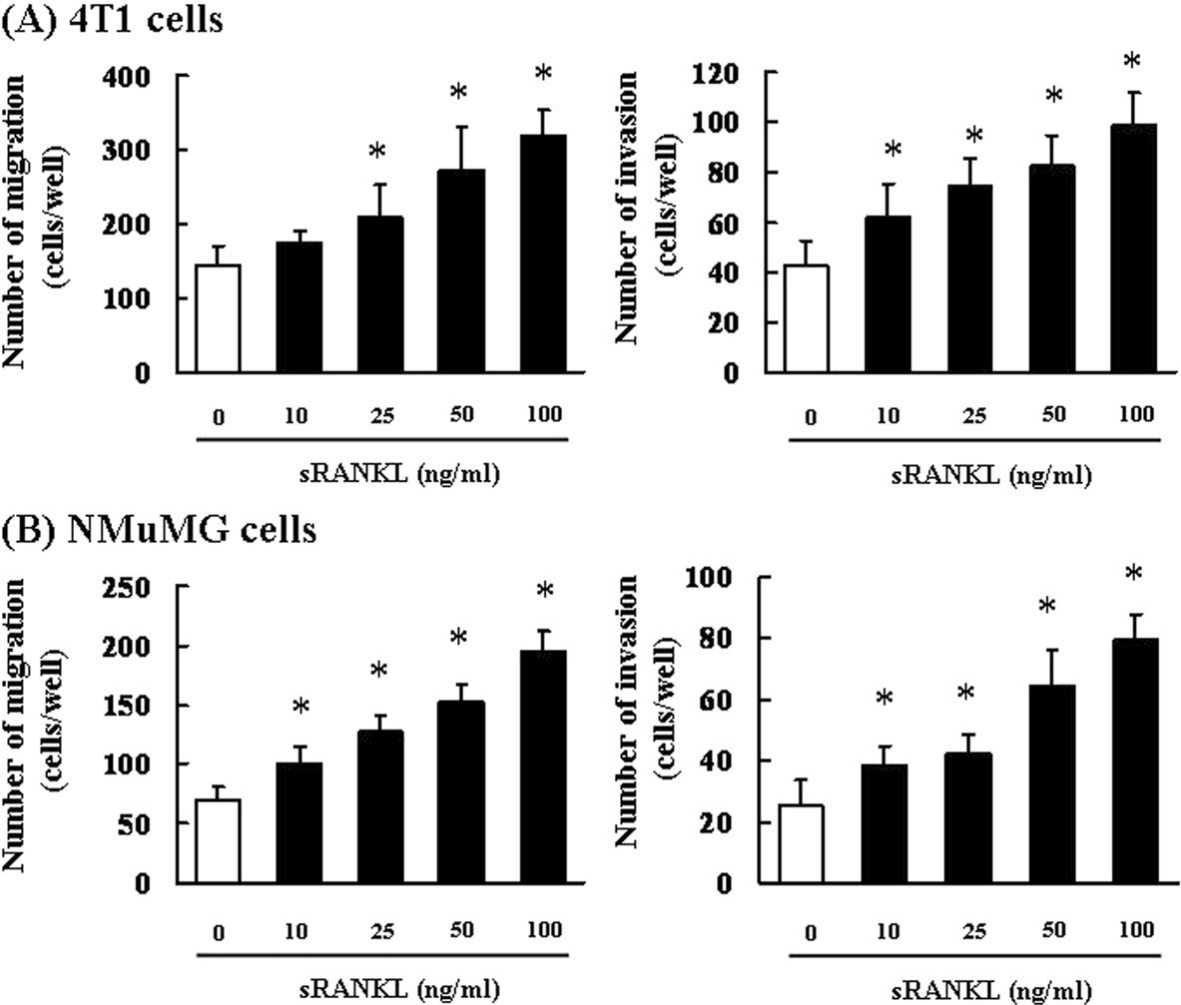
**RANKL-induced EMT promotes cell migration and invasion. (A)** 4T1 cells and **(B)** NMuMG cells were pretreated with 10, 25, 50, or 100 ng/mL RANKL for 24 h, after which 5 × 10^3^ cells were seeded into the upper compartments of chambers. Migration was analyzed using Boyden chamber assays with Falcon cell culture inserts. Invasive properties were analyzed using Falcon cell culture inserts covered with 50 μg of Matrigel per filter. For both assays, the lower chambers contained conditioned media (serum-free medium with the addition of RANKL), which was used as a chemoattractant. After incubation for 24 h, the cells invading the lower surface were counted microscopically. The results are representative of 5 independent experiments. *p < 0.01 vs. controls (ANOVA with Dunnet’s test).

Considering the effect of RANKL-mediated EMT of breast cancer cells and normal mammary epithelial cells, we next examined its role in cell migration and invasion, which accompany EMT, using the Boyden chamber and Matrigel invasion chamber assays, respectively. Upon RANKL treatment, the number of 4T1 and NMuMG cells migrating and invading through the chambers significantly increased in a concentration-dependent manner (Figure 2A–2B). Furthermore, small interfering RNA-mediated silencing of RANK expression suppressed RANKL-induced cell migration and invasion (data not shown).

These results indicate that RANKL plays an essential role in the regulation of breast cancer cells through the induction of EMT.

### RANKL-mediated epithelial-mesenchymal transition in breast cancer cells and normal mammary epithelial cells is dependent on NF-κB signaling

**Figure 3:**
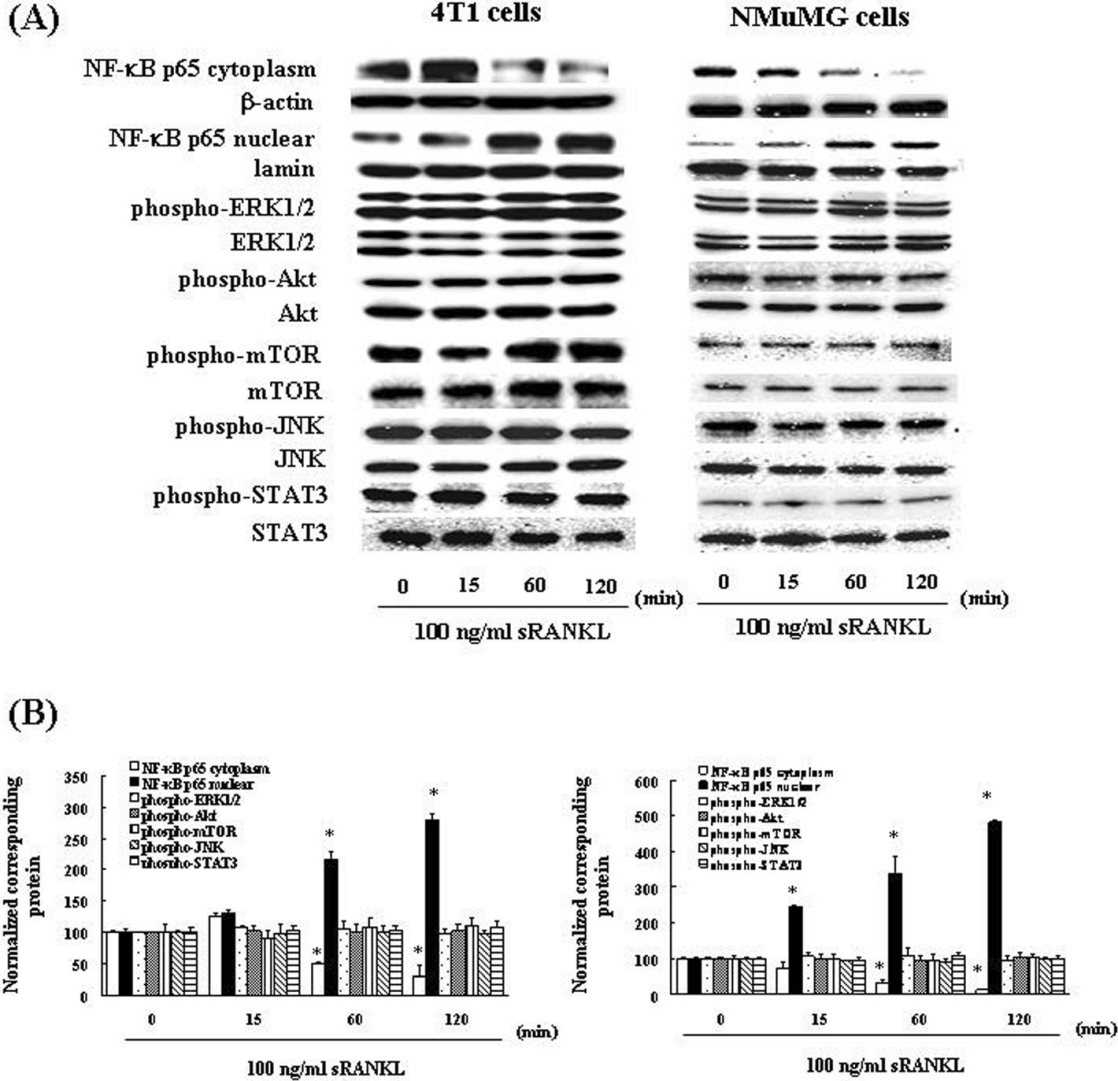
**RANKL induces the activation of NF-κB. (A)** 4T1 and NMuMG cells were incubated with 100 ng/mL RANKL. At various time points, the cytoplasmic fractions and nuclear fractions were extracted and then subjected to SDS-PAGE/immunoblotting with anti-NF-κB p65, anti-phospho-ERK1/2, anti-phospho-Akt, anti-phospho-mTOR, anti-phospho-JNK, anti-phospho-STAT3, anti-ERK1/2, anti-Akt, anti-mTOR, anti-JNK, and anti-STAT3 antibodies. Anti-β-actin and anti-lamin antibodies were used as internal standards. **(B)** Quantification of the amount of NF-κB p65, phospho-ERK1/2, phospho-Akt, phospho-mTOR or phospho-STAT3, normalized to the amounts of the corresponding proteins, respectively. The results are representative of 5 independent experiments. *p < 0.01, compared to controls (ANOVA with Dunnett’s test).

In order to investigate which signaling pathways are induced when RANKL induces EMT in 4T1 and NMuMG cells, we examined the changes that occur in the localization of NF-κB p65 and phosphorylation of ERK 1/2, Akt, mTOR, JNK, and STAT3 after the addition of RANKL. In 4T1 and NMuMG cells, unlike the control cells, the degree of nuclear localization of the NF-κB p65 subunit was found to increase when examined at 60 and 120 min after RANKL stimulation (Figure 3). On the other hand, the amount of the NF-κB p65 subunit localized in the cytoplasm decreased at 60 and 120 min after RANKL stimulation (Figure 3). Using the control cells as reference, we observed no substantial changes in the levels of ERK1/2, Akt, mTOR, JNK, and STAT3 phosphorylation (Figure 3).

**Figure 4:**
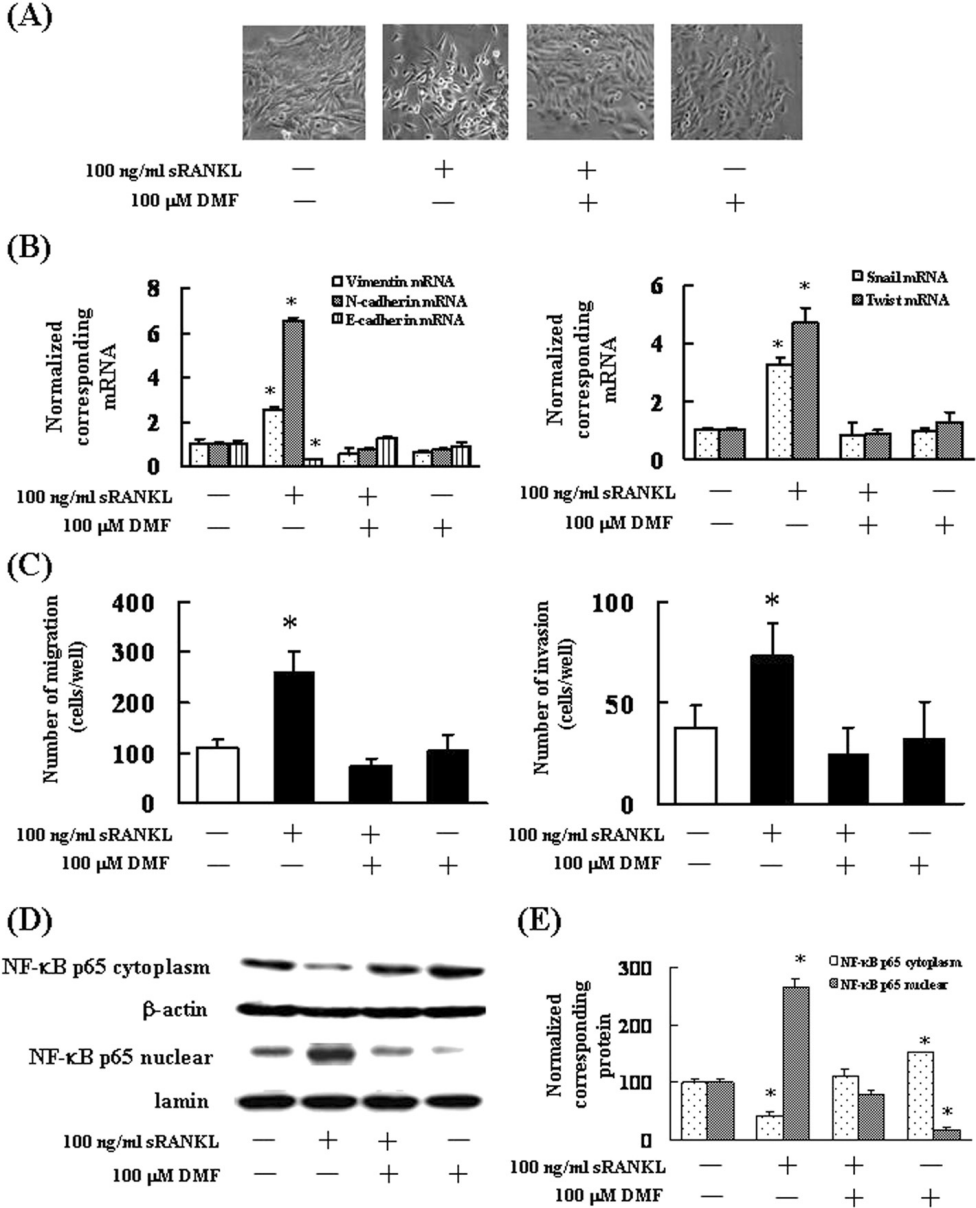
**Effects of DMF on RANKL-induced EMT and EMT-related mRNA expression. (A)** Analysis of 4T1 cell morphology after cell treatment of with 100 ng/mL RANKL or 100 μM DMF (× 40 magnification). **(B)** Total RNA was extracted, and the mRNA levels of vimentin, E-cadherin, N-cadherin, Snail, and Twist were determined by real-time PCR. The results are expressed as treated over control ratio after correction to GAPDH mRNA levels. The results are representative of 5 independent experiments. *p < 0.01, as compared to controls (ANOVA with Dunnett’s test). **(C)** 4T1 cells were pretreated with 100 ng/mL RANKL or 100 μM DMF for 24 h, after which 5 × 10^3^ cells were seeded into the upper compartments of chambers. Migration was analyzed by Boyden chamber assays using Falcon cell culture inserts. Invasive properties were analyzed using Falcon cell culture inserts covered with 50 μg of Matrigel per filter. For both assays, the lower chambers contained conditioned media (addition of RANKL in serum-free medium), which was used as a chemoattractant. After incubation for 24 h, the cells invading the lower surface were counted microscopically. The results are representative of 5 independent experiments. *p < 0.01 vs. the controls (ANOVA with Dunnet’s test). **(D)** 4T1 cells were incubated with 100 ng/mL RANKL or 100 μM DMF. After 60 min, the cytoplasmic fractions and nuclear fractions were extracted and then subjected to SDS-PAGE/immunoblotting with anti-NF-κB p65 antibody. Anti-β-actin and anti-lamin antibodies were used as the internal standard. **(E)** Quantification of the amount of NF-κB p65, normalized to the amounts of the corresponding proteins, respectively. The results are representative of 5 independent experiments. *p < 0.01, as compared to controls (ANOVA with Dunnett’s test).

Thus far, the results indicate that RANKL-mediated EMT in 4T1 and NMuMG cells occurs via activation of the NF-κB p65 subunit. Therefore, we treated 4T1 cells with DMF, a NF-κB inhibitor, in order to determine whether suppression of the NF-κB p65 subunit would result in the inhibition of RANKL-mediated EMT. Administration of DMF inhibited the RANKL-mediated changes in the morphology of 4T1 cells (Figure 4A). Next, we investigated whether DMF suppressed the RANKL-mediated upregulation of EMT markers, cell migration, and invasion. DMF inhibited the upregulation of EMT markers, cell migration, and invasion in 4T1 cells (Figure 4B–4C). In addition, DMF suppressed the nuclear translocation of NF-κB by RANKL stimulation (Figure 4D–4E). These results indicate that NF-κB plays an essential role in the RANKL/RANK system.

## Discussion

In this study, we demonstrated that RANKL induces EMT through the upregulation of Snail and Twist expression levels in normal breast epithelial cells and breast cancer cells. We also found that RANKL-induced EMT accelerated cell migration and invasion in normal breast epithelial cells and breast cancer cells. It has been indicated that aberrant RANK signaling promotes breast tumorigenesis [20]. It has also been reported that RANKL induces the migration and metastasis of RANK-expressing cancer cells [16-18]. In addition, high RANK expression levels in primary tumors of patients have been correlated with poor prognoses and higher risk of developing bone metastasis [21]. Collectively, the findings suggest that the RANKL/RANK system promotes cell migration, invasion, and metastasis by EMT in RANK-expressing cancer cells.

RANKL/RANK signaling activates a variety of downstream pathways. RANK assembles into functional trimers. Various tumor necrosis factor receptor-associated factor proteins associate with the cytoplasmic domain of RANK and mediate ligand-induced signaling. RANKL/RANK induces the activation of NF-κB mediated by the I-κB kinase complex [22,23]. Members of the mitogen-activated protein kinase family, including JNK and ERK, are activated downstream of RANK [24,25]. RANK also induces the activation of the phosphoinositol 3-kinase/Akt/mTOR pathway and the Janus kinase 2/STAT3 pathway [26,27]. Our results clearly demonstrate that RANKL induces activation of NF-κB but not of ERK1/2, Akt, mTOR, JNK, and STAT3. It has been reported that the activation of NF-κB upregulated the expression levels of Snail and fibronectin and induced EMT [28,29]. It has also been indicated that NF-κB activation promotes cell migration and invasion by stabilization of Snail in breast cancer cells [30]. Furthermore, it has been reported that NF-κB-induced Twist expression required EMT in normal breast epithelial cells and breast cancer cells [31]. Collectively, these results suggest that RANKL/RANK signaling induces EMT by NF-κB activation and upregulation of Snail and Twist in normal breast epithelial cells and breast cancer cells. Moreover, we observed that DMF, a NF-κB inhibitor, inhibited RANKL-induced EMT and enhanced the expressions of Snail and Twist, cell migration, and invasion. A previous report has shown that NPI-0052, a proteasome inhibitor, suppresses EMT via the inhibition of NF-κB activation and Snail expression [32]. It has also been reported that inhibition of the NF-κB signaling pathway suppresses tumor necrosis factor α-induced EMT and Twist expression [31]. In addition, these results indicate that a decrease in the activation of NF-κB induced by DMF in breast cancer cells plays an important role in the inhibition of EMT, Snail and Twist expression, migration, and invasion.

Breast cancer often invades bone tissue, causing skeletal complications due to metastasis [33]. In more than 75% of all breast cancer patients, bone metastasis was found at the time of autopsy [34]. EMT is the first step that allows the extravasation and migration of carcinoma cells in the metastatic process. EMT entails the downregulation of E-cadherin and the upregulation of its suppressor, Snail and Twist, in carcinoma cells [5,6,10]. Resent studies showed that Twist was frequently observed in the bone marrow of breast cancer patients and the expression of Twist correlated with the rapid occurrence of distant metastasis or local progression [35]. It has been indicated that Snail-positive breast cancer tends to home into the bone in breast cancer patients [36]. In addition, more than 80% of bone metastases from solid tumors, including carcinoma and sarcoma, are RANK-positive, as revealed by immunohistochemistry [17,21]. Moreover, it has been reported that inhibition of RANKL by recombinant osteoprotegerin, a decoy receptor for RANKL, suppressed tumor bone metastasis and progression and improved survival in a mouse model [37]. The present results clearly indicated that the RANKL/RANK system induced EMT via enhanced expression of Snail and Twist, and the activation of NF-κB. Collectively, these findings suggest that RANKL-induced EMT may play an important role in bone metastasis in RANK-expressing cancer cells.

## Conclusion

In conclusion, our data show that RANKL induces EMT, cell migration, and invasion through the activation of NF-κB and upregulation of Snail and Twist. These findings suggest that the RANKL/RANK system promotes tumor cell migration, invasion, and metastasis via the induction of EMT.

## Abbreviations

EMT: Epithelial-mesenchymal transition; RANK: Receptor activator of nuclear factor-κB; RANKL: RANK ligand; NF-κB: Nuclear factor-κB; DMF: Dimethyl fumarate; PBS: Phosphate buffer saline; GAPDH: Glyceraldehyde-3-phosphate dehydrogenase; NP-40: Nonyl phenoxylpolyethoxylethanol; SDS: Sodium dodecyl sulfate; PVDF: Polyvinylidene fluoride; ERK: Extracellular signal-regulated kinase; mTOR: mammalian target of rapamycin; JNK: c-Jun N-terminal kinase; STAT3: Signal transducers and activator of transcription 3.

## Competing interests

The authors declare that they have no competing interests.

## Authors’ contributions

MT carried out analysis of EMT, western blotting analysis, real time PCR, migration and invasion assays, statistical analysis, and drafted the manuscript. MK and SF carried out analysis of EMT, western blotting analysis. TI, TS, MI, KS, and HS carried out western blotting analysis. TT, NO, KM, and DF carried out migration and invasion assays. JM, KS, and TS contributed to statistical analyses. SN designed the experiments and revised the manuscript. All authors read and approved the final manuscript.
